# Grain-Sized Moxibustion Heightens the AntiTumor Effect of Cyclophosphamide in Hepa1-6 Bearing Mice

**DOI:** 10.1155/2022/3684899

**Published:** 2022-08-08

**Authors:** Tao Zhu, Yanzhu Ma, Jianyun Wang, Xiaolin Chen, Jianhao Li, Liqiang Meng, Yuduo Hou, Yanting Cheng

**Affiliations:** ^1^Shanxi University of Chinese Medicine, Jinzhong 030600, China; ^2^Department of Infectious Diseases, First Affiliated Hospital of Zhengzhou University, Zhengzhou 450052, China

## Abstract

**Objective:**

The side effects of chemotherapy as a treatment of liver cancer cannot be ignored. Grain-sized moxibustion, a characteristic external therapy, has been shown to reduce the toxic and side effects of chemotherapy and regulate the immune function. The purpose of this study was to explore the synergistic antitumor activity of grain-sized moxibustion combined with cyclophosphamide (CTX).

**Methods:**

A hepatoma 1–6 (Hepa1-6)-bearing mouse model was established by injecting mice with Hepa1-6 cancer cells. CTX and grain-sized moxibustion on Dazhui (DU14), Zusanli (ST36), and Sanyinjiao (SP6) were used for treatment, and mouse survival status, body weight, and tumor growth, weight, and volume were measured. White blood cells (WBCs) and bone marrow nucleated cells (BMNCs) were quantified. The spleens and livers of Hepa1-6-bearing mice were pathologically examined and scored. Serum aspartate aminotransferase (AST) and alanine aminotransferase (ALT) levels were measured with enzyme-linked immunosorbent assay (ELISA) kits, and protein and mRNA expression levels of Ki67 and proliferating cell nuclear antigen (PCNA) in tumor tissues were measured with immunohistochemistry and real-time quantitative polymerase chain reaction (RT-qPCR) techniques.

**Results:**

Both grain-sized moxibustion and CTX could restrain the growth of Hepa1-6 tumors, reducing both tumor volume and weight; the combined treatment had a greater effect. Grain-sized moxibustion down-regulated the expression of proliferation genes Ki67 and PCNA, weakened the proliferation ability of Hepa1-6 tumor cells, inhibited tumor growth, and enhanced the antitumor effect of CTX. In addition, grain-sized moxibustion significantly improved the signs of CTX-induced toxicity (including weight loss, leukopenia, bone marrow suppression, and hepatotoxicity), down-regulated serum AST and ALT levels, reduced spleen and liver inflammation, and improved liver and spleen indices.

**Conclusion:**

Grain-sized moxibustion can synergize with CTX to enhance the antitumor effect of CTX and alleviate its toxic and side effects. It may be a promising adjuvant therapy to chemotherapy.

## 1. Introduction

Hepatocellular carcinoma is one of the most common malignant tumors worldwide, with rapid progression and high mortality [1, 2]. In the clinic, chemotherapy is still an important treatment for inoperable liver cancer or postoperative recurrence of liver cancer [3, 4]. Cyclophosphamide (CTX), as an alkylating agent that selectively targets tumor cells, is significant in the treatment of malignant tumors through releasing nitrogen from phosphoramidase to inhibit liver cancer [5].

Although CTX inhibits tumor cells, it also causes certain damage to normal cells. One example of this damage is the inhibition of myeloid cell differentiation, resulting in decreased blood cells, damage to immune cells, and a decrease in body immune resistance [6–8]. Therefore, chemotherapy is of vital importance to treat malignant tumors, but its side effects are still a medical problem. It is necessary to find new chemotherapy adjuvant therapies or drugs that reduce toxicity and enhance antitumor activity to achieve better efficacy.

Grain-sized moxibustion, a characteristic external therapy, can directly stimulate specific acupoints to treat diseases through the photothermal stimulation of specific acupoints in a short period of time. It plays the roles of dredging the meridians, regulating qi and blood, strengthening the body, and eliminating the pathogens [9, 10]. In terms of adjuvant chemotherapy in the treatment of malignant tumors, reports on moxibustion can be traced back to the 1970s. For example, many clinical studies have shown that moxibustion can treat leukopenia [11]. Subsequently, we carried out the screening of moxibustion and acupoint prescriptions and conducted relevant basic experimental research [12]. Based on the above, this study aimed to determine whether grain-sized moxibustion can reduce the toxic and side effects of CTX and explored whether it can synergistically inhibit the liver cancer.

Thus, this study investigated the effect of grain-sized moxibustion alone or in combination with CTX on the hepatoma 1–6 (Hepa1-6)-bearing mouse model to evaluate the antitumor activity of grain-sized moxibustion in vivo in an effort to identify potential clinical treatment options.

## 2. Materials and Methods

### 2.1. Animals

Forty healthy and clean C57BL/6J male mice (4–6 weeks old, body weight 16–18 g, *n* = 40) were purchased from Beijing Vital River Laboratory Animal Technology Co., Ltd. (Beijing, China) (Animal laboratory license number: SCXK Beijing 2016–0006).

All the mice were raised in the Key Laboratory of the First Affiliated Hospital of Zhengzhou University, with a light-dark cycle of 12/12 hours, an ambient temperature of 22–25°C, a humidity of 45 ± 10%, and free feeding and drinking. In addition, all operations on mice were authorized according to ethical regulations and the Animal Care Ethics Committee of Shanxi University of Chinese Medicine (License number: 2019DW233).

### 2.2. Cell Culture

Hepa1-6 cells were obtained from the Cell Bank of the Chinese Academy of Sciences (Shanghai, China) and cultured in Dulbecco's modified Eagle medium (DMEM; Gibco, USA) containing 10% fetal bovine serum (FBS; Gibco, USA) and 1% penicillin/streptomycin (Gibco, USA) at 37 °C in a 5% CO_2_ humid environment.

After the frozen Hepa1-6 cells were removed from liquid nitrogen, they were continuously shaken in a 37°C water bath to promote their thawing. They were next moved into a 15 mL centrifuge tube and 10 mL preheated complete DMEM medium was added. The cells were gently blown into a suspension and centrifuged at 2000 rpm for 2 minutes and the supernatant was discarded. Ten milliliters DMEM were added to wash the pellet and the supernatant was discarded. Ten milliliters complete DMEM were added to the cells, which were gently blown into a suspension and cultured in a 5% CO_2_ cell incubator. When the cell density of Hepa1-6 cells reached 80–90%, the medium was removed and the cells were washed twice with 10 mL phosphate-buffered saline (PBS). Three milliliters trypsin containing 0.25% ethylenediaminetetraacetic acid (EDTA) was added to the cell culture flask for 3 minutes. One milliliter complete DMEM was added to stop trypsin digestion and the cells were transferred to a 15 mL centrifuge tube. Ten milliliters complete DMEM were added to a clean cell culture plate. The cells transferred to the 15 mL centrifuge tube were centrifuged at 2000 rpm for 2 minutes and the supernatant was discarded. Ten milliliters PBS were added to create the cell suspension and 10 *μ*l of the suspension was removed for counting cells using the cell counting plate. The cells were plated according to 3 × 10^6^ cells/mL inoculation.

### 2.3. Model Establishment and Treatment

**Figure 1:**
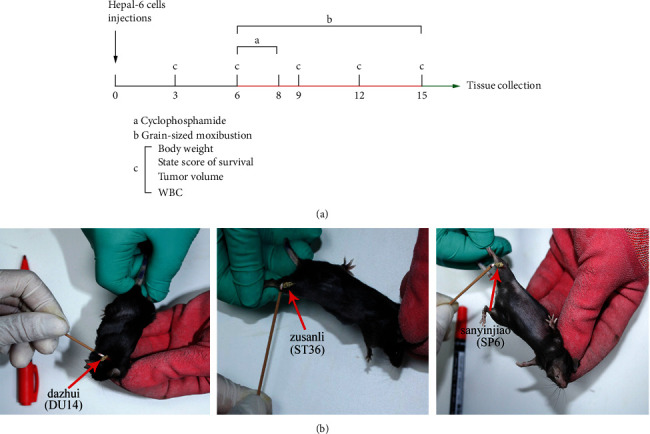
Schematic diagram of the experimental procedure and mouse acupoints. (a) Flowchart of experimental interventions. a. Cyclophosphamide (CTX), (b) Grain-sized moxibustion, c. Body weight, state score of survival, tumor volume, white blood cell (WBC). The Hepa1-6-bearing mice in the T and GT groups were treated with CTX on days 6, 7, and 8. The tumor-bearing mice in the G and the GT groups were treated with moxibustion on days 6–15 (once a day). The tumor volume, state of survival, and WBCs were measured on days 3, 6, 9, 12, and 15, and mice were sacrificed on day 16 for tissue collection. b. The location of acupoints (Dazhui DU14, Zusanli ST36, and Sanyinjiao SP6, red arrow) in Hepa1-6-bearing mice. c. Control group; g. grain-sized moxibustion group; T: CTX group, GT: grain-sized moxibustion plus CTX group.

After a week of adaptation, all mice were randomly divided into four groups (*n* = 10 each), which included the control group (C), grain-sized moxibustion group (G), CTX group (T), and grain-sized moxibustion plus CTX group (GT). The density of the Hepa1-6 tumor cells was adjusted to 3 × 10^6^ cells/mL [13], and 0.2 mL was extracted and inoculated subcutaneously into right armpit of C57BL/6 mice to establish the Hepa1-6-bearing mouse model [14]. As seen in in Figure 1(a), five days after injection tumors were palpable (approximately 50 mm^3^ in size). Hepa1-6-bearing mice in the T and the GT groups were administered CTX (Baxter Oncology GmbH, Germany, Approval number: 9L353 A) at 30 mg/kg by intraperitoneal injection on the 6^th^, 7^th^, and 8th day [15], whereas mice in the C group were administered an equivalent volume (0.2 mL) 0.9% sodium chloride by intraperitoneal injection [16]. From day 6 to day 15, mice in the G and GT groups were treated with grain-sized moxibustion upon three chosen acupoints (Figure 1(a)): Dazhui (DU14, unilateral), Zusanli (ST36, bilateral), and Sanyinjiao (SP6, bilateral). Five moxa cones were placed at each acupoint once a day (the G and the GT groups were treated with grain-sized moxibustion at the same time every day). The size and operation methods of the grain-sized moxibustion were as follows [17–19]: after the mice were immobilized, the moxibustion acupoints were depilated. The moxa velvet (Nanyang Kangaiduo Wormwood Products Co., Ltd. Nanyang, Henan, China) was made into a moxa cone the size of a grain of wheat (about 0.5 g/grain), which was placed to burn on the acupoint where the petroleum jelly (Vaseline, Unilever, London, United Kingdom) was applied. When the moxa cone burned to 3/4, the remaining moxa cone was quickly removed with tweezers. The next one was then lit.

On day 16, mice in each group were weighed and anesthetized with ether, and blood samples were drawn from the medial canthus of each mouse. After mice were sacrificed with cervical dislocation, the tumor, spleen, and liver tissues were collected under sterile conditions. The residual blood was then removed with filter paper and accurately weighed. The visceral index, tumor volume, and tumor inhibition rate were calculated. The formulas were as follows: visceral index = organ weight/body weight, tumor volume (mm^3^) = (length × width^2^)/2 [20], and tumor inhibition rate (%) = (average tumor mass in the control group−average tumor mass in the treatment group)/average tumor mass of the control group × 100% [21].

The body weights of mice in each group were measured at 9:00 a.m. on days 3, 6, 9, 12, and 15 of the experiment. The survival status of each mouse was observed and scored. Survival status scores are shown in Table S1 [22].

### 2.4. Biochemical and Hematological Analysis

On the 3rd, 6th, 9th, 12th, and 15th days of the experiment, the tail vein blood of each tumor-bearing mouse was drawn to observe the changes in the number of white blood cells (WBCs). After treatment, blood was collected from the intraocular canthus of the Hepa1-6-bearing mice and put into an EDTA anticoagulation tube. Serum alanine aminotransferase (ALT) and aspartate aminotransferase (AST) levels of Hepa1-6-bearing mice in each group were determined by enzyme-linked immunosorbent assay (ELISA) kits (AST 13320-1-AP, ALT 16757-1-AP; Proteintech, USA). In addition, the right femurs of Hepa1-6-bearing mice in each group were taken, and the bone marrow cavity of each femur was repeatedly washed with PBS buffer until it turned white. The bone marrow cell suspension was resuspended, and the number of bone marrow nucleated cells (BMNCs) was counted using a light microscope (CX33; Olympus, Tokyo, Japan) after lysis of the red blood cells.

### 2.5. Histological Analysis

Formalin-fixed spleen and liver tissues were dehydrated in graded ethanol solutions, transparent in xylene, immersed in paraffin, and then embedded in conventional paraffin to make wax blocks. The tissue wax blocks were cut into tissue slices 3–5 *μ*m thick and then adhered to the tissue sections. They were dewaxed in xylene, rehydrated in gradient alcohol, immersed in hematoxylin (*H*) for 3 minutes, differentiated, returned to blue, and washed with pure water. Eosin (*E*) was added dropwise for 20 s; the tissue was washed with pure water, dehydrated with graded ethanol solutions, cleared in xylene for 10 minutes, and sealed with neutral gum. Pathological changes were observed using a light microscope (CX33; Olympus, Tokyo, Japan). The pathology scores are as shown in Tables S2 and S3 [23, 24].

### 2.6. Immunohistochemistry to Detect Ki67 and PCNA Protein Expression

The tumor tissues were fixed with 4% paraformaldehyde and then made into paraffin blocks. Subsequently, the tissue wax block was cut into tissue sections with a thickness of about 5 *μ*m, dewaxed, and rehydrated with pure water for washing. Sodium citrate (pH 6.0) was used for microwave antigen retrieval at 98°C. The sections were then cooled down to room temperature, incubated in 3% hydrogen peroxide solution for 10 minutes, and blocked with 3% bovine serum albumin (BSA) for 50 minutes. The tumor tissue sections were individually incubated with 50 *μ*L primary antibodies (1 : 500 anti-Ki67 antibodies, 1 : 1,000 anti-PCNA antibodies; Wuhan Servicebio Biotechnology Co., Ltd. Wuhan, China) overnight at 4°C. The next day the tumor tissue sections were incubated with 50 *μ*L HRP-labeled secondary antibody (goat anti-rabbit IgG, 1 : 200; Wuhan Servicebio Biotechnology Co., Ltd. Wuhan, China) for 50 minutes at room temperature, washed with PBS three times for 10 min each, and subsequently incubated with diaminobenzidine for color development. After the nuclei were stained with hematoxylin, the sections were dehydrated, cleared, and mounted with neutral gum. Immunohistochemistry of the tumor tissues was observed using a light microscope (CX33; Olympus, Tokyo, Japan). Images were captured using a microscope, and PCNA and Ki67 expression were evaluated by counting the number of positive cells from three randomly selected fields in the residual viable tumor tissue among the necrotic areas using a light microscope at magnifications of 200× and 400×. Digital images were then analyzed with Image J to evaluate the percentage of positive area for all antibodies used in the immunohistochemistry analyses [25, 26].

### 2.7. Real-Time Quantitative Polymerase Chain Reaction (RT-qPCR) to Detect Ki67 and PCNA mRNA Expression

After the tumor tissues were fully crushed, the total RNA of tumor tissue was extracted with a Trizol kit (Wuhan Servicebio Biotechnology Co., Ltd. Wuhan, China), and its concentration and purity were detected with a UV-Vis spectrophotometer (ES-2, Malcom, Tokyo, Japan). The reverse transcription kit synthesized cDNA from the RNA. Primer design and synthesis were completed by Shanghai Bioengineering Co., Ltd. Total mRNA was used as a template for reverse transcription into cDNA for real-time fluorescence quantitative PCR amplification in a 20 *μ*L system. The reaction system was as follows: 1 *μ*L qPCR primer, 1 *μ*L cDNA product, and 10 *μ*L SYBR Green qPCR Master Mix (2×). Amplification conditions: pre-denaturation 95°C for 10 min followed by 40 cycles of 95°C for 15 s and 60°C for 60 s. The melting curve is completed at 60°C for 95°C, and the temperature was increased by 0.3°C every 15 s. Glyceraldehyde-3-phosphate dehydrogenase was used as an endogenous reference, and the 2^−ΔΔCt^ method was used to analyze and calculate the relative expression of the target gene. The primer sequences are shown in Table S4.

### 2.8. Statistical Analysis

All data were entered into SPSS 25.0 software (IBM SPSS Inc., Chicago, IL, USA) for analytical processing and testing for homogeneity of variance and normality. All data were expressed as mean ± SD. *P* < 0.05 was considered statistically significant. One-way analysis of variance was used for eligible groups of three or more. If the variances were unequal, Dunnett's T3 method was used. If the data did not conform to a non-normal distribution, the nonparametric Kruskal–Wallis test was used.

## 3. Results

### 3.1. The Effect of Grain-Sized Moxibustion Combined with CTX on Tumor Growth in Hepa1-6-Bearing Mice

**Figure 2:**
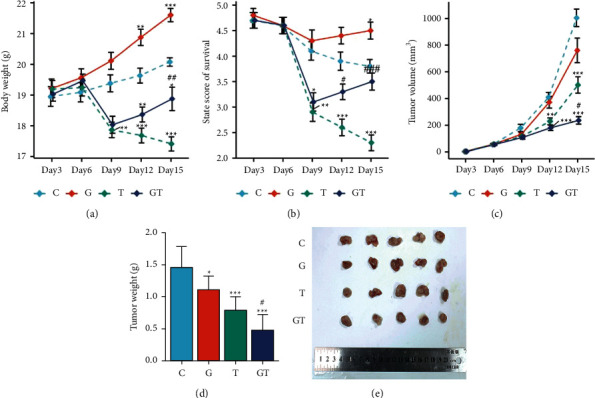
The effect of grain-sized moxibustion combined with CTX on tumor growth in Hepa1-6-bearing mice. (a) Body weight (days 6, 9, 12, and 15). (b) State score of survival (days 6, 9, 12, and 15). (c) Tumor volume (days 6, 9, 12, and 15). (d) Tumor weight. (e) Tumor photograph. All values are presented as means ± SD (*n* = 10). Notes: ^*∗*^*P* < 0.05, ^*∗∗*^*P* < 0.01, ^*∗∗∗*^*P* < 0.001 vs. C group; ^#^*P* < 0.05, ^##^*P* < 0.01 vs. T group. C: control group; G: grain-sized moxibustion group; T: CTX group; GT: grain-sized moxibustion plus CTX group.

To observe whether grain-sized moxibustion can heighten the antitumor effect of CTX, we evaluated changes in body weight, survival status, and tumor growth curves in Hepa1-6 bearing mice treated with grain-sized moxibustion, CTX, and grain-sized moxibustion combined with CTX. As shown in Figure 2(a) and 2(b), the survival status of Hepa1-6-bearing mice in each group was higher before the intervention; the difference was not statistically significant. However, survival status of the Hepa1-6-bearing mice in the T and GT groups deteriorated significantly on the 9th day compared with that of the C group (*P* < 0.01 and *P* < 0.05, respectively). The body weight of the Hepa1-6-bearing mice in the T group was also greatly reduced (*P* < 0.01), which may be due to the toxicity of CTX. On day 12, the Hepa1-6-bearing mice in group C had a slow weight gain and poor survival status, which may be due to cachexia. Compared with the C group, the body weight of Hepa1-6-bearing mice in the G group was significantly higher (*P* < 0.01) and the survival status was improved, whereas the weight of Hepa1-6-bearing mice in the T and GT groups was significantly lower (*P* < 0.001 and *P* < 0.01, respectively). Compared with the T group, survival status of the Hepa1-6-bearing mice in the GT group was significantly higher (*P* < 0.05). On the 15th day, the weight of Hepa1-6-bearing mice in the G group was significantly higher compared with the C group (*P* < 0.001). The survival status was significantly improved (*P* < 0.05), whereas the weight of Hepa1-6-bearing mice in the T and GT groups was significantly lower (*P* < 0.001 and *P* < 0.05, respectively), especially in the T group. The survival status of the Hepa1-6-bearing mice was significantly lower compared to C group (*P* < 0.001). Compared with the T group, the weight of the Hepa1-6-bearing mice in the GT group was significantly higher (*P* < 0.01) and the survival status also significantly improved (*P* < 0.001). In addition, the difference in tumor volume of the Hepa1-6-bearing mice in each group (5 days after tumor cell transplantation) was not statistically significant. As shown in Figure 2(c), on day 12 (1 week after intervention, 2 weeks after tumor cell transplantation), compared with group C, the tumor volume of Hepa1-6 tumor-bearing mice in group T and GT decreased significantly (*P* < 0.01 and *P* < 0.001, respectively). On the 15th day, the tumor volume of the Hepa1-6-bearing mice in the G group was lower than that in the C group, but the difference was not statistically significant. The tumor volume of the Hepa1-6-bearing mice in the T group and the GT group was significantly lower than that in the C group (both *P* < 0.001). It is worth noting that Hepa1-6-bearing mice in the GT group experienced a stronger inhibitory effect on the tumor growth than did the T group, and the tumor volume was significantly lower than that in the C group (*P* < 0.05). Similar results were observed in images of tumors and tumor weights of mice on day 16 (Figures 2(d) and 2(e)). In addition, the tumor inhibition rate in group G was 23.9%, in group T was 45.9%, and in group GT was 67.1%, indicating that grain-sized moxibustion could heighten the antitumor effect of CTX.

### 3.2. The Effect of Grain-Sized Moxibustion in Combination with CTX on Spleen and Liver Tissue of Hepa1-6-Bearing Mice

**Figure 3:**
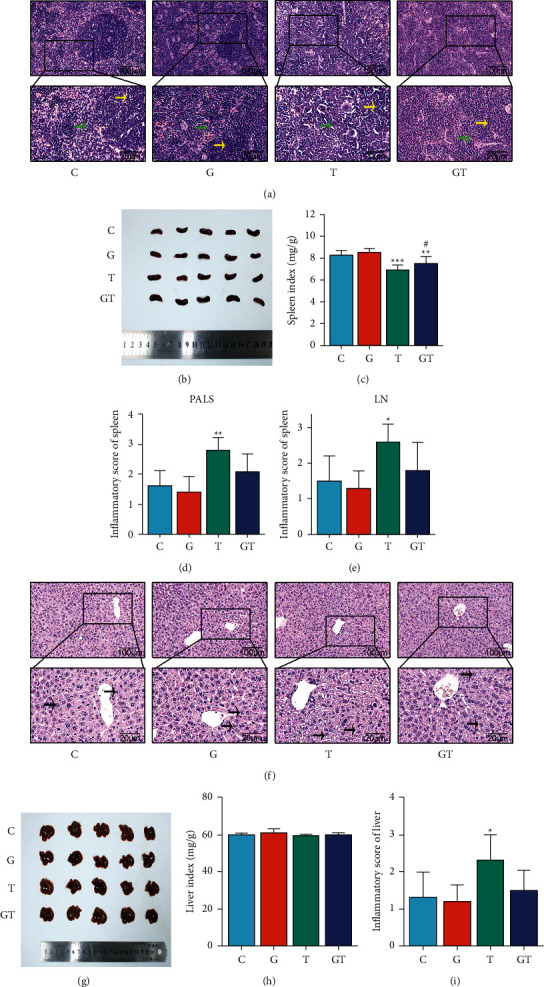
The effect of grain-sized moxibustion combined with CTX on spleen and liver tissue of Hepa1-6-bearing mice. (a) H and E staining of the spleen (×100, ×400). (b) Spleen photograph. (c) Spleen index. (d) Inflammatory score of periarteriolar lymphoid sheath (PALS). (e) Inflammatory score of lymphoid nodule (LN). (f) H&E staining of the liver (×100, ×400). (g) Liver photograph. (h) Liver index. (i) Inflammatory score of liver. All values are presented as means ± SD (*n* = 10). PALS as yellow arrow), LN as green arrow, and Hepatocyte as black arrow. Notes: ^*∗*^*P* < 0.05, ^*∗∗*^*P* < 0.01, ^*∗∗∗*^*P* < 0.001 vs. C group; ^#^*P* < 0.05, ^##^*P* < 0.01 vs. T group. C: control group; G: grain-sized moxibustion group; T: CTX group; GT: grain-sized moxibustion plus CTX group.

To determine whether grain-sized moxibustion combined with CTX affects the spleen and liver weights in Hepa1-6-bearing mice, spleen and liver indices were calculated. As shown in Figure 3(c), the spleen index of the mice in the G group was higher than that of the C group, but there was no statistical significance. The spleen index of the mice in the T group and the GT group was significantly lower than that of the C group (*P* < 0.001 and *P* < 0.01, respectively). Compared with the T group, the spleen index of the GT group was significantly higher (*P* < 0.05). It is worth noting that the differences in the liver indices among the four groups were not significant, suggesting that the grain-sized moxibustion combined with CTX may have little influence on liver weight.

Next, to further explore whether the grain-sized moxibustion can improve the organ damage caused by CTX in Hepa1-6-bearing mice, H&E staining was performed on the spleens and livers of Hepa1-6-bearing mice in each group. Pathomorphological changes were observed (100×, 400×), and inflammation scores were recorded.

See Figure 3(a) shows a histological micrograph of the spleen. In group C, a clear boundary between the red and white pulp was observed and the cells were neat and dense. A clear boundary between the red and white pulp was also observed in the G group. The density of periarteriolar lymphoid sheath (PALS) and lymphoid nodule (LN) cells was not significantly different than that of group C. In the T group, the boundary between red and white pulp was unclear and the density of PALS and LN cells increased (*P* < 0.01 and *P* < 0.05, respectively). However, cells in the GT group were denser than those in the T group and the boundary of the red pulp and the white pulp was clearer. This indicated that grain-sized moxibustion could reduce the density of cells in the PALS and the proliferation of LN cells, suggesting that grain-sized moxibustion could reduce CTX-induced spleen damage in Hepa1-6-bearing mice.

See Figure 3(f) shows microscopic pictures of liver histology. In the C group, the structures of hepatocyte nuclei were clear and the cell infiltration was low. The nuclei of hepatocytes in the G group were clearly displayed. There was no obvious abnormal change in the cell structure. In comparison to group C, hepatocytes in group T were severely degenerated and swollen, cell boundaries were blurred or even absent, inflammatory cells infiltrated around the central vein, and the cellular inflammation was distinct (*P* < 0.05). Compared with the T group, the hepatocyte structures of the GT group were different and the degree of cell necrosis and inflammation was milder, but the difference was not statistically significant. The results suggest that grain-sized moxibustion could alleviate the pathological changes of liver tissues caused by CTX. Changes in liver structures may be caused by the toxicity of CTX rather than moxibustion. Notably, the infiltration of inflammatory cells in the C group may be induced by Hepa1-6 cancer cells.

### 3.3. The Effect of Grain-Sized Moxibustion in Combination with CTX on Blood and Biochemical/Hematological (or Cellular) Indexes in Hepa1-6-Bearing Mice

**Figure 4:**
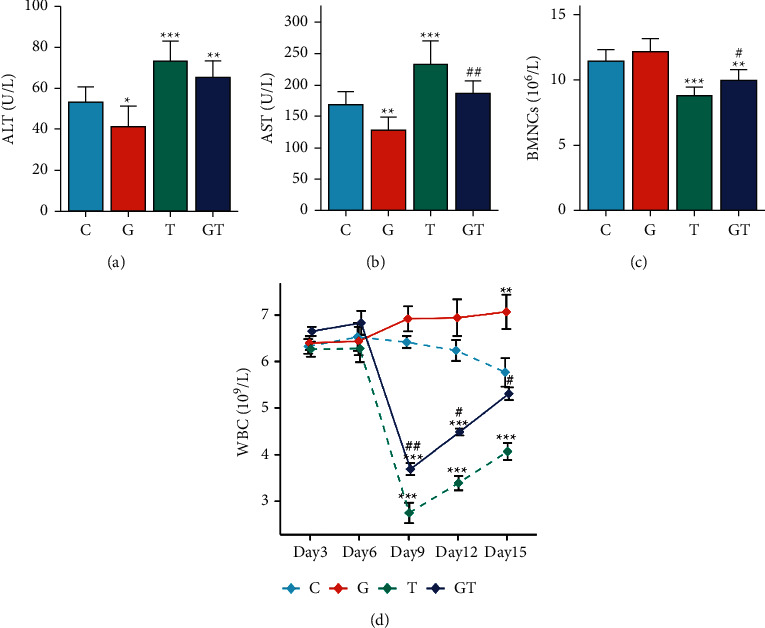
The effect of grain-sized moxibustion combined with CTX on hematological and biochemical indexes of Hepa1-6-bearing mice. (a) ALT: alanine aminotransferase. (b) AST: aspartate transaminase. (c) BMNCs: bone marrow nucleated cells. (d) WBCs: white blood cells (days 6, 9, 12, and 15). All values are presented as means ± SD (*n* = 10). Notes: ^*∗*^*P* < 0.05, ^*∗∗*^*P* < 0.01, ^*∗∗∗*^*P* < 0.001 vs. C group; ^#^*P* < 0.05, ^##^*P* < 0.01 vs. T group. C: control group; G: grain-sized moxibustion group; T: CTX group, GT: grain-sized moxibustion plus CTX group.

Biochemical indicators are important biological indicators to characterize the experimental animals [27]. In comparison to those in group C, serum ALT and AST levels in group G were significantly lower (*P* < 0.05 and *P* < 0.01, respectively) (Figures 4(a) and 4(b)). Serum ALT and AST levels in the T group were significantly higher than those of group C (both  *P* < 0.001). In the GT group, serum ALT levels were significantly higher than those of group C (*P* < 0.01), whereas serum AST levels were higher but not statistically significant. Compared with those in the T group, serum AST levels in the GT group were significantly lower (*P* < 0.01). Serum ALT levels were lower but not statistically significant. These results indicate that grain-sized moxibustion could significantly improve the liver injury caused by CTX, which was consistent with the results of liver inflammation scoring.

As shown in Figure 4(d), the numbers of WBCs in groups T and GT were significantly lower than those in group C on the 9th day (both  *P* < 0.001). Compared with those in the T group, the numbers of WBCs in the GT group were significantly higher (*P* < 0.01). On the 12th day, the numbers of WBCs in groups T and GT were significantly lower than those in group C (both  *P* < 0.001). In comparison to those in group T, the numbers of WBCs in group GT were significantly higher (*P* < 0.05). On day 15, the numbers of WBCs in group T were significantly lower in comparison to group C (*P* < 0.001). Compared with those in the T group, the numbers of WBCs in the GT group were significantly higher (*P* < 0.05). Notably, the number of WBCs in the T group increased slowly after day 9, indicating that the inhibitory effect of CTX on WBCs may be short-lived. In addition, we measured the number of BMNCs in Hepa1-6-bearing mice. The results showed that the number of BMNCs in the G group was higher than that of the C group, but not statistically significant, whereas the numbers of BMNCs in the T and GT groups were significantly lower (*P* < 0.01 and  *P* < 0.001, respectively). Compared with those of the T group, the number of BMNCs in GT group was significantly higher (*P* < 0.05). These findings indicate that grain-sized moxibustion could improve bone marrow suppression induced by CTX.

### 3.4. The Effect of Grain-Sized Moxibustion in Combination with CTX on Tumor Cell Proliferation in Hepa1-6-Bearing Mice

**Figure 5:**
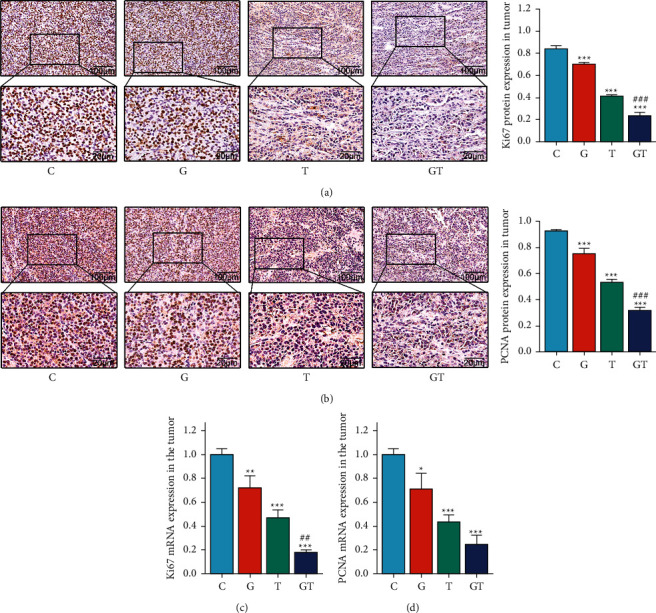
The effect of grain-sized moxibustion combined with CTX on the proliferation of tumor cells in Hepa1-6-bearing mice. (a) Expression of Ki67 protein in tumor tissue. (b) Expression of PCNA protein in tumor tissue. (c) The relative expression of Ki67 mRNA in tumor tissue. (d) The relative expression of PCNA mRNA in tumor tissue. All values are presented as means ± SD (*n* = 3). Notes. ^*∗*^*P* < 0.05, ^*∗∗*^*P* < 0.01, ^*∗∗∗*^*P* < 0.001 vs. C group; ^#^*P* < 0.05, ^##^*P* < 0.01 vs. T group. C: control group; G: grain-sized moxibustion group; T: CTX group, GT: grain-sized moxibustion plus CTX group.

To assess the proliferative activity of tumor cells and observe whether the inhibitory effect of grain-sized moxibustion combined with CTX on tumor growth in mice is related to the proliferation genes Ki67 and PCNA, we performed immunohistochemical analysis of tumor proliferation activity. Compared with those in the C group, the numbers of Ki67-and PCNA-positive cells in tumor tissues of the G, T, and GT groups were lower (Figures 5(a) and 5(b)) and the Ki67 and PCNA protein expression levels were significantly lower (all  *P* < 0.001). Compared with those in group T, the number of Ki67-and PCNA-positive cells in tumor tissues of group GT was lower and the Ki67 and PCNA protein expression levels were significantly lower (both  *P* < 0.001).

Based on the above results, we carried out RT-qPCR analysis to further clarify the mechanism of grain-sized moxibustion combined with CTX in vivo. As shown in Figures 5(c) and 5(d), the relative mRNA expression levels of Ki67 and PCNA in group G were significantly lower after treatment with grain-sized moxibustion in comparison to those in group C (*P* < 0.01 and *P* < 0.05, respectively). The relative mRNA expression levels of Ki67 and PCNA in the T and GT groups were significantly lower than those of group C (all *P* < 0.001). However, the relative mRNA expression levels of Ki67 in the GT group were significantly lower compared with those of the T group (*P* < 0.01); the relative mRNA expression levels of PCNA were lower, but it had no statistical significance. These results suggest that grain-sized moxibustion inhibited tumor growth by down-regulating the expression of proliferation genes Ki67 and PCNA, consistent with the results of immunohistochemical analysis. Likewise, the combined application of grain-sized moxibustion and CTX could enhance the antitumor effects, inhibit the proliferation of tumor cells, and exert a synergistic effect.

## 4. Discussion

This study evaluated the effect of grain-sized moxibustion and CTX on tumor growth, weight, volume, and inhibition rate. The body weight and survival status of Hepa1-6-bearing mice were significantly decreased after CTX treatment. However, after treatment with grain-sized moxibustion, the weight and survival status of Hepa1-6-bearing mice in the GT group were significantly higher than those in the T group. Furthermore, the results showed that both grain-sized moxibustion and CTX could effectively inhibit tumor growth.

The combined use of grain-sized moxibustion and CTX had a synergistic effect, which could significantly increase the tumor inhibition rate. CTX is one of the most effective and widely used drugs in the synthesis of many chemotherapeutic drugs. It works mainly through the replacement of nitrogen mustard methyl groups with CTX rings to form intra- and inter-strand DNA cross-links, thereby interfering with DNA replication and inhibiting tumor growth [28, 29]. Compared with other therapies, grain moxibustion has unique advantages in treating cancer [30, 31]. In recent years, relevant basic research and clinical trials have shown that moxibustion can increase the number of lymphocytes and their subsets, activate natural killer cells, and stimulate the activity of cytokines (e. g., interleukin (IL)-1 and IL-2) to improve the body's immune response [32] and the survival status of patients with cancer [33]. These findings are consistent with our experimental results. At present, there is no research on whether grain-sized moxibustion combined with CTX has a synergistic effect on the treatment of tumors. This study observed synergistic effects of grain-sized moxibustion on Hepa1-6-bearing mice and the effect of reducing toxicity of chemotherapy drugs (e. g., CTX).

It is well known that the most common clinical adverse reactions caused by CTX are leukopenia [34], bone marrow suppression [35], and hepatotoxicity [36]. It also has a certain impact on the hematopoietic system [37]. The hematopoietic system is one of the most sensitive targets of toxic compounds and is often regarded as an important parameter of physiological and pathological states in humans and animals [38]. To study the relevant mechanism, we observed the effect of grain-sized moxibustion and CTX on WBCs and BMNCs and conducted related research. The results showed that grain-sized moxibustion combined with CTX could significantly increase the numbers of WBCs and BMNCs compared with those of CTX alone and had a certain protective effect on the hematopoietic system. Serum ALT and AST are sensitive markers to detect whether liver function is reduced, which can reflect different degrees of liver damage [39]. Grain-sized moxibustion can significantly reduce serum ALT and AST levels and alleviate CTX-induced hepatotoxicity. Organ weight is one of the most sensitive indicators of drug toxicity, and changes in organ weights often precede morphological changes [10]. This study found that grain-sized moxibustion could significantly reduce the damage to the liver and spleen caused by CTX and increase the liver and spleen indices. It has been reported that the cancer usually occurs at sites of chronic inflammation [40]. Moreover, inflammatory cells are also involved in the formation of tumors and impair immunity. Remarkably, the density of PALS cells in the spleen was lower, the proliferation of LN was inhibited, and the degeneration and swelling of hepatocytes were lower in the GT group than in the C group. Grain-sized moxibustion could significantly improve CTX-induced splenic lymphocyte reduction and reduce liver inflammation, thereby improving the pathological changes of spleen and liver and exerting an immunoregulatory effect. Overall, grain-sized moxibustion combined with CTX could significantly improve the survival status of Hepa1-6-bearing mice, increase body weight, improve symptoms such as leukopenia, myelosuppression, and hepatotoxicity, reduce the organ inflammatory response, and improve liver and spleen indices. These findings are consistent with the research of some domestic scholars, specifically that moxibustion could reduce the adverse reactions and damage to the body induced by chemotherapy [41].

Ki67 is a nuclear protein associated with cellular proliferation [42], and the quantity of nuclear staining cells is associated with tumor stage and disease development [43]. PCNA is a protein involved in the replication and repair of DNA and the adjustment of the cell cycle [44] and plays an important role in DNA replication and maintaining gene stability [45]. Ki67 and PCNA were shown to be strongly expressed in stained cells in previous investigations, implying that tumor cells were active and proliferated rapidly [46]. Consequently, Ki67 and PCNA could reflect the proliferative activity of tumor cells, suggesting their potential status as prognostic indicators. In this study, Ki67 and PCNA were greatly overexpressed in group C as shown by many brown-yellow particles in the nucleus, indicating that the tumor cells were in an active stage and a bad prognosis. The expression levels of Ki67 and PCNA in the G and T groups were significantly lower than those in the C group, whereas the expression levels of Ki67 and PCNA in the GT group were lower than those in the T group. The results of immunohistochemistry and RT-qPCR confirmed those of the other, indicating that the combination therapy could enhance the antitumor activity accompanied by an improved prognosis. Furthermore, our results showed that grain-sized moxibustion could assist CTX in the inhibition of tumor cell proliferation. The inhibitory effect is more obvious with time and its survival time is predicted to be prolonged, which may be the significance of grain-sized moxibustion as adjuvant chemotherapy.

This study preliminarily investigated the effect of grain-sized moxibustion on the antitumor effects of CTX in Hepa1-6-bearing mice and provided new insights into the antitumor effects of grain-sized moxibustion and the reduction of the toxic and side effects of CTX. Nonetheless, this study has limitations such as a short observation time and small sample size. To further study the therapeutic mechanism of grain-sized moxibustion, the effect of grain-sized moxibustion on tumor cell apoptosis can be further observed and studied by extending the course of treatment and increasing the sample size. Hence, we anticipate that relevant basic research and clinical follow-up studies will improve our experimental conclusions.

## 5. Conclusion

The antitumor effect of grain-sized moxibustion combined with CTX on Hepa1-6-bearing mice is greater than that of CTX alone. The mechanism by which grain-sized moxibustion enhances the antitumor activity of CTX may include restraining tumor cell proliferation and reducing the toxicity of CTX. Thus, grain-sized moxibustion may be a promising external therapy for adjuvant chemotherapy.

## Data Availability

The data used to support the findings of this study are included within the article.

## Abbreviations

Hepa1-6: Hepatoma 1–6
CTX: Cyclophosphamide
FBS: Fetal bovine serum
BSA: Bovine serum albumin
AST: Aspartate aminotransferase
ALT: Alanine aminotransferase
BMNCs: Bone marrow nucleated cells
WBC: White blood cell
PALS: Periarteriolar lymphoid sheath
LN: Lymphoid nodule
RT-qPCR: Real-time quantitative polymerase chain reaction.

## Supplementary Materials

Supplementary Materials Table S1: Survival status scores of tumor-bearing mice in this study.

## References

[1] Lokau J., Schoeder V., Haybaeck J., Garbers G. (2019). Jak-stat signaling induced by interleukin-6 family cytokines in hepatocellular carcinoma. *Cancers*.

[2] Wang J., Zhao H., Yu J. (2021). MiR-320b/RAD21 axis affects hepatocellular carcinoma radiosensitivity to ionizing radiation treatment through DNA damage repair signaling. *Cancer Science*.

[3] Liu Q., Yu X., Yang M. (2020). A study of the mechanism of lncRNA-CR594175 in regulating proliferation and invasion of hepatocellular carcinoma cells in vivo and in vitro. *Infectious Agents and Cancer*.

[4] Zhang Y., Xie C., Li A. (2019). PKI-587 enhances chemosensitivity of oxaliplatin in hepatocellular carcinoma through suppressing DNA damage repair pathway (NHEJ and HR) and PI3K/AKT/mTOR pathway. *Ameican Journal of Translational Research*.

[5] Podgurskaya A. D., Slotvitsky M. M., Tsvelaya V. A. (2021). Cyclophosphamide arrhythmogenicity testing using human-induced pluripotent stem cell-derived cardiomyocytes. *Scientific Reports*.

[6] Shi M., Zhang J., Li X. (2018). Mitochondria-targeted delivery of doxorubicin to enhance antitumor activity with HER-2 peptide-mediated multifunctional pH-sensitive DQAsomes. *International Journal of Nanomedicine*.

[7] Huang Y., He Y., Ye S., Zhong Q., Chen Z. (2013). Combined use of cyclophosphamide and Chalone 19-peptide in experimental breast cancer. *Oncology Targets and Therapy*.

[8] Amiri F., Hamzeh M., Hosseinimehr S., Khalatbary A., Mohammadi H., Dashti A. (2018). Atorvastatin mitigates cyclophosphamide-induced hepatotoxicity via suppression of oxidative stress and apoptosis in rat model. *Research in Pharmaceutical Sciences*.

[9] Zhang Y., Li J., Wei D. (2020). Genome-wide regulation of electroacupuncture and treadmill exercise on diet-induced obese rats. *Evidence-Based Complementary and Alternative Medicine*.

[10] Wen J., Zhuang Z., Zhao M. (2018). Treatment of poststroke constipation with moxibustion: a case report. *Medicine (Baltimore)*.

[11] Ma M. (1994). TCM treatment and research progress of leukopenia. *Sichuan TCM*.

[12] Cheng Y. T., Hou Y. D. (2016). Literature quality evaluation of randomized controlled trials on acupuncture and moxibustion for secondary leukopenia in China. *Journal of Shanxi University of Traditional Chinese Medicine*.

[13] Liu Z., Lu Z., Jing R. (2019). Alarmin augments the antitumor immunity of lentiviral vaccine in ectopic, orthotopic and autochthonous hepatocellular carcinoma mice. *Theranostics*.

[14] Liu P., Zhao L., Pol J. (2019). Crizotinib-induced immunogenic cell death in non-small cell lung cancer. *Nature Communications*.

[15] Farouk M. M., El-Molla A., Salib F. A., Soliman Y. A., Shaalan M. (2020). The role of silver nanoparticles in a treatment approach for multidrug-resistant Salmonella species isolates. *International Journal of Nanomedicine*.

[16] Wang Z., Xu X. Y., Li Q. (2017). Anti-tumor effect of ginsenoside Rg1 pyrolysis products (HPPRg1) on H22 tumor-bearing mice. *Chinese Journal of Pharmacy*.

[17] Zhang X., Wan Q., Xu T. S. (2017). Effect of grain-moxibustion on IL-6 and STAT 3 in inflammatory microenvironment of lewis lung cancer mice. *Acupuncture Research*.

[18] Wang W. J., Wan X. X., Xu T. S. (2014). Effect of moxa-grain-moxibustion on serum Th1/Th2 type cytokines in lewis tumor-bearing mice. *Acupuncture Research*.

[19] Hu D., Shen W., Gong C. (2021). Grain-sized moxibustion promotes NK cell antitumour immunity by inhibiting adrenergic signalling in non-small cell lung cancer. *Journal of Cellular and Molecular Medicine*.

[20] Naito S., von Eschenbach A. C., Giavazzi R., Fidler I. J. (1986). Growth and metastasis of tumor cells isolated from a human renal cell carcinoma implanted into different organs of nude mice. *Cancer Research*.

[21] Wang L., Wang R. (2015). Effect of rapamycin (RAPA) on the growth of lung cancer and its mechanism in mice with A549. *International Journal of Clinical and Experimental Pathology*.

[22] Tan J., Yang R. D., Zhao H., Zhuojun P., Lizhi O., Yaping L. (2019). Inhibitory effect of moxibustion on tumor growth in rats with gastric tumor. *World Science and Technology-Modernization of Traditional Chinese Medicine*.

[23] Gao M., Zhang M., Si M., Chen J.-Y., Wei W. (2018). The expression of CXCL12/CXCR4 in spleen of adjuvant arthritis rats and the effect of paeoniflorin-6′-O-benzene sulfonate. *China Pharmacology Circular*.

[24] She Y. L., Yan D. Q., Liu Y. Q. (2014). Effect of danggui beimu kushen pill on pathological morphology of tumor and liver, kidney and thymus of H22 tumor-bearing mice treated with cisplatin. *Chinese Journal of TCM Information*.

[25] Metawea O. R. M., Abdelmoneem M. A., Haiba N. S. (2021). A novel “smart” PNIPAM-based copolymer for breast cancer targeted therapy: synthesis, and characterization of dual pH/temperature-responsive lactoferrin-targeted PNIPAM-co-AA. *Colloids and Surfaces B: Biointerfaces*.

[26] Wang Y., Deng X., Yu C. (2018). Synergistic inhibitory effects of capsaicin combined with cisplatin on human osteosarcoma in culture and in xenografts. *Journal of Experimental & Clinical Cancer Research*.

[27] Chen X., Yang H., Wang Z. (2019). The effect of different dietary levels of defatted rice bran on growth performance, slaughter performance, serum biochemical parameters, and relative weights of the viscera in geese. *Animals*.

[28] Emadi A., Jones R. J., Brodsky R. A. (2009). Cyclophosphamide and cancer: golden anniversary. *Nature Reviews Clinical Oncology*.

[29] van Dorp W., Mulder R. L., Kremer L. C. (2016). Recommendations for premature ovarian insufficiency surveillance for female survivors of childhood, adolescent, and young adult cancer: a report from the international late effects of childhood cancer guideline harmonization group in collaboration with the PanCareSurFup consortium. *Journal of Clinical Oncology*.

[30] El-Far A. H., Godugu K., Noreldin A. E. (2021). Thymoquinone and costunolide induce apoptosis of both proliferative and doxorubicin-induced-senescent colon and breast cancer cells. *Integrative Cancer Therapies*.

[31] Khafaga A. F., Shamma R. N., Abdeen A. (2021). Celecoxib repurposing in cancer therapy: molecular mechanisms and nanomedicine-based delivery technologies. *Nanomedicine*.

[32] Pei J., Wei H., Liu Z. D., Yu Y. M., Ni C. R., Wu H. G. (2010). Effects of moxibustion on the expression of IL-1beta, IL-2, IL-6 mRNA and protein in the cerebral cortex in tumor-bearing mice. *Acupuncture Research*.

[33] Wang P., Zhu J., Xie X. L. (2016). Effects on the tumor specific growth factor and tumor necrosis factor *α* in rats’ precancerous lesion of primary hepatocellular carcinoma by direct moxibustion at Ganshu (BL 18) acupoint. *Chinese Journal of Integrative Medicine*.

[34] Ma L., Wang Y., Bo J. (2016). Autologous cytokine-induced killer (CIK) cell immunotherapy combined with cyclophosphamide in five patients with POEMS syndrome. *Clinical and Experimental Immunology*.

[35] Evans K., Duan J., Pritchard T. (2019). OBI-3424, a novel AKR1C3-activated prodrug, exhibits potent efficacy against preclinical models of T-ALL. *Clinical Cancer Research*.

[36] Zhao M., Zhao H., Deng J., Guo L., Wu B. (2019). Role of the CLOCK protein in liver detoxification. *British Journal of Pharmacology*.

[37] Ding Z. C., Lu X., Yu M. (2014). Immunosuppressive myeloid cells induced by chemotherapy attenuate antitumor CD4+ T-cell responses through the PD-1-PD-L1 axis. *Cancer Research*.

[38] Belemkar S., Shendge P. N. (2021). Toxicity profiling of the ethanolic extract of *Citrullus lanatus* seed in rats: behavioral, biochemical and histopathological aspects. *Bioscience Reports*.

[39] Chen Z., Zhang F., Jiang L., Chen Z., Sun H. (2021). Toxic effects of mycotoxin fumonisin B1 at six different doses on female BALB/c mice. *Toxins*.

[40] Liu Y., Zhang L., Zhu X., Wang Y., Liu W., Gong W. (2015). Polysaccharide Agaricus blazei Murill stimulates myeloid derived suppressor cell differentiation from M2 to M1 type, which mediates inhibition of tumour immune-evasion via the Toll-like receptor 2 pathway. *Immunology*.

[41] Zhang H. W., Lin Z. X., Cheung F., Cho W. C., Tang J. L. (2018). Moxibustion for alleviating side effects of chemotherapy or radiotherapy in people with cancer. *Cochrane Database of Systematic Reviews*.

[42] Othman E. M., Bekhit A. A., Anany M. A., Dandekar T., Ragab H. M., Wahid A. (2021). Design, synthesis, and anticancer screening for repurposed pyrazolo[3, 4-d] pyrimidine derivatives on four mammalian cancer cell lines. *Molecules*.

[43] Chandrasekaran B., Pal D., Kolluru V. (2018). The chemopreventive effect of withaferin A on spontaneous and inflammation-associated colon carcinogenesis models. *Carcinogenesis*.

[44] Okamoto H., Muraki I., Okada H. (2021). Recombinant antithrombin attenuates acute respiratory distress syndrome in experimental endotoxemia. *American Journal of Pathology*.

[45] Kamikawa Y., Yokota K., Oikawa K., Sato F., Muragaki Y. (2020). Suppression of MKL1 promotes adipocytic differentiation and reduces the proliferation of myxoid liposarcoma cells. *Oncology Letters*.

[46] Wang Q. S., Gao L. N., Zhu X. N. (2019). Co-delivery of glycyrrhizin and doxorubicin by alginate nanogel particles attenuates the activation of macrophage and enhances the therapeutic efficacy for hepatocellular carcinoma. *Theranostics*.

