# Supplementary material for: Grain-Sized Moxibustion Heightens the AntiTumor Effect of Cyclophosphamide in Hepa1-6 Bearing Mice
Source: Evid Based Complement Alternat Med. 2022 Aug 8;2022:3684899. doi: 10.1155/2022/3684899 (PMC9377901; doi:10.1155/2022/3684899)
Supplement: Supplementary Materials — Table S1: Survival status scores of tumor-bearing mice in this study. [file 3684899.f1.zip › 3684899.f1/TableS1.docx]

Table S1. Survival status scores of tumor-bearing mice in this study

| Survival status manifestation | score |
| --- | --- |
| Normal tumor-bearing mice, free movement, bright hair color. | 5 |
| Steady breathing, more sensitive to external stimuli, able to stand and walk, brighter coat color but slower gait. | 4 |
| Conscious, could crawl autonomously, part of the mice hairs were shed, and the hairs were rough and yellow. | 3 |
| Lethargic, unresponsive, shallow and fast breathing, more mouse hair loss, rougher hair. | 2 |
| Coma, lying still, no obvious response to external stimuli, or extreme sluggishness. | 1 |
| Death | 0 |
