# Supplementary material for: Grain-Sized Moxibustion Heightens the AntiTumor Effect of Cyclophosphamide in Hepa1-6 Bearing Mice
Source: Evid Based Complement Alternat Med. 2022 Aug 8;2022:3684899. doi: 10.1155/2022/3684899 (PMC9377901; doi:10.1155/2022/3684899)
Supplement: Supplementary Materials — Table S1: Survival status scores of tumor-bearing mice in this study. [file 3684899.f1.zip › 3684899.f1/TableS2.docx]

Table S2: Spleen pathology scores of tumor-bearing mice in this study

| Periarteriolar lymphoid sheath (PALS) | Lymphoid nodule (LN) | Score |
| --- | --- | --- |
| Normal. | Normal. | 0 |
| Slightly increased cell density. | Slight hyperplasia with visible germinal center. | 1 |
| Cell density increased moderatelywith crowding. | Moderate hyperplasia with obvious germinal centers. | 2 |
| Cell density increased severely with overlapping cells. | Excessive hyperplasia with more frequent germinal centers. | 3 |
