# Supplementary material for: Grain-Sized Moxibustion Heightens the AntiTumor Effect of Cyclophosphamide in Hepa1-6 Bearing Mice
Source: Evid Based Complement Alternat Med. 2022 Aug 8;2022:3684899. doi: 10.1155/2022/3684899 (PMC9377901; doi:10.1155/2022/3684899)
Supplement: Supplementary Materials — Table S1: Survival status scores of tumor-bearing mice in this study. [file 3684899.f1.zip › 3684899.f1/TableS3.docx]

Table S3：Liver pathological scores

| Histopathological manifestation | Score |
| --- | --- |
| Hepatocytes were neatly arranged, with normal shape, clear lobular structure, and no pathological changes. | 0 |
| Hepatocyte swelling, ballooning, and punctate necrosis. | 1 |
| Focal necrosis of hepatocytes. | 2 |
| Extensive focal necrosis of hepatocytes. | 3 |
