# Supplementary material for: Grain-Sized Moxibustion Heightens the AntiTumor Effect of Cyclophosphamide in Hepa1-6 Bearing Mice
Source: Evid Based Complement Alternat Med. 2022 Aug 8;2022:3684899. doi: 10.1155/2022/3684899 (PMC9377901; doi:10.1155/2022/3684899)
Supplement: Supplementary Materials — Table S1: Survival status scores of tumor-bearing mice in this study. [file 3684899.f1.zip › 3684899.f1/TableS4.docx]

Table S4：The gene-specific primers used in this study.

| Gene | Ki67 | PCNA | GAPDH |
| --- | --- | --- | --- |
| Primer information | NM 001081117.2 | NM 011045.2 | NM 008084.2 |
| Forward primer (5′-3′) | AATCTGTGGAAGAGCAGGTTAGC | GAGCAACTTGGAATCCCAGAAC | CCTCGTCCCGTAGACAAAATG |
| Reverse primer (5′-3′) | TCCTGGGAGGCAGTCTTCATAG | ACCGCCTCCTCTTCTTTATCCA | TGAGGTCAATGAAGGGGTCGT |
| Product size | 199 | 215 | 133 |
| Annealing temperature (°C) | Forward:60.62 Reverse:60.96 | Forward:59.51 Reverse:60.89 | Forward:59.7 Reverse:61.5 |
| GC% | Forward:47.83 Reverse:54.55 | Forward:50 Reverse:50 | Forward:52.4 Reverse:52.4 |
| Number of bases | Forward:23 Reverse:22 | Forward:52.4 Reverse:52.4 | Forward:21 Reverse:21 |
